# Pleiotropic Immune Functions of Chemokine Receptor 6 in Health and Disease

**DOI:** 10.3390/medicines5030069

**Published:** 2018-07-02

**Authors:** Ranmali Ranasinghe, Rajaraman Eri

**Affiliations:** School of Health Sciences, College of Health and Medicine, University of Tasmania, Launceston, Tasmania 7250, Australia; ranmaliranasinghe301@gmail.com

**Keywords:** CCR6, CCL20, T_H_17, T_reg_, inflammation, multiple organs, health and disease

## Abstract

C-C chemoattractant cytokine (chemokine) receptor 6 (CCR6) and its exclusive binding molecule CCL20 is an extremely important chemokine receptor-ligand pair which controls cell migration and immune induction during inflammatory disease. Not many scientific studies have been undertaken to study its immune mechanisms in detail, but its unique contribution to steady state cell chemotaxis in upholding immune tolerance and regulating immune homeostasis during inflammation is evident in multiple systems in the human body, including skin, liver, lung, kidney, brain, eye, joints, gonads and the gut. The role of CCR6 is constitutively expressed as a series of much debilitating severe inflammatory and autoimmune diseases, Human Immunodeficiency Virus (HIV) and cancer metastasis. CD4^+^ T cells, the central organizers of adaptive immunity, are stringently mobilized by the CCR6/CCL20 axis also induced by cytokines and a host of other factors in a carefully executed immune modulation scenario, to bring about a delicate balance between inflammation inducing T_H_17 cells and regulatory T_reg_ cells. Although the exact immune regulatory role is not elucidated as yet, the CCR6/CCL20 axis is implicated as a front runner which determines the polarization of T_H_17 and regulatory T_reg_ cells, upon which depends the resolution or progression of many debilitating disorders. This review therefore aims at emphasizing the pleiotropic significance of the chemokines CCR6 and CCL20 in immunologic function in multiple organ systems, thereby hoping to accentuate its value in future therapeutic modalities.

## 1. Introduction

### Chemokines

Chemokines represent an exclusive cell directing system in the body, consisting of signaling proteins vital to the immune system. The primary role of chemokines in infections is to attract immune cells bearing their cognate receptors to sites of inflammation. The best example is corroborated by the role of chemokines in mucosal immunity, where epithelial cells of the mucosa activated by an inflammatory stimulus releases the chemokine ligands, constitutively inducing chemotaxis of the leukocytes bearing their corresponding receptors towards them [1]. This is necessarily how chemokines mediate immune modulation and maintain cell migration during immune homeostasis or inflammation. Chemokines have also been described by other names, such as a specific ‘cell positioning’ system [2] or a potential ‘cell navigating’ system [1] which brings about immune modulation in multiple organ systems of the human body.

The chemokine repertoire described so far includes 50 different chemokines and 20 different receptors. Chemokine receptors are externally displayed on the surface of a range of cells [2] such as T and B lymphocytes, macrophages, dendritic cells, peripheral blood mononuclear cells, innate lymphoid cells, neuron, epithelial and endothelial cells [3].

Chemokines, abbreviated from ‘chemoattractant cytokines’, mediate their biological effects across G-protein coupled receptors [1]. These surface–bound receptors are specifically borne by a wide range of immune and non-immune cells. Biochemically, chemokine receptors function as guanine nucleotide exchange factors, mainly limited to the pertussis–toxin sensitive G1 class of G proteins. Their immunological effects consist of coordinating leucocyte development, differentiation, distribution, chemotactic migration and their effector capabilities [4]. The biological impact of chemokines produces multiple outcomes: (i) embryonic development; (ii) angiogenesis; (iii) T-helper subset development; (iv) leukocyte homeostasis; (v) wound healing; (vi) lymph organogenesis; (vii) inflammatory disorders; (viii) tumor proliferation and metastasis; and (ix) B cell maturation and antigen-induced B cell differentiation [1,4,5,6,7,8].

Chemokines consist of small proteins having a molecular weight of 8 to 14 kilo Dalton [9]. The structure reveals three beta pleated sheets with an alpha helix at the carbon terminal and disulfide bonds connecting cysteine subunits. Chemokines are said to be composed of secreted, structurally related proteins having 67–127 amino acids. The functional form is highlighted as a monomer, although they could exist as either a monomer, dimer or tetramer [1]. Chemokines are named based on their receptors and each sub class, CC, CXC, CX3C or XC is identified by the number and spacing of preserved cysteines. R denotes receptor and it is followed by a number which lists out the chronological order of discovery [9]. Thus “CC chemokines have CCR chemokine receptors” to bind with. Some chemokines bind specifically to one receptor such as the cognate receptor, CCR6 and its exclusive ligand, CCL20. Chemokines can ligate with more than one receptor or several chemokine receptors can attach to more than one chemokine, among which the CCR6 and its ligand CCL20 forms an exclusive monogamous pair [1]. Chemokines are known to actively recruit immature or effector cells to affected sites during maintenance of immune tolerance or promoting inflammation, but interestingly inhibition of chemokines have shown to develop anti-inflammatory properties, encouraging the exploration of novel therapeutic breakthroughs in medical treatment [10].

Chemokine-aided T cell chemotaxis is directly linked to a number of immune-activated diseases. Leukocyte migration from blood to tissue is a multi-step process. It involves selectin-mediated rolling on endothelium and chemoattractant-mediated integrin activation, followed by leukocyte extravasation and chemotaxis up a chemoattractant gradient [4]. Upon synthesis within the cell, chemokines are secreted and get tethered to glycosaminoglycans, a group of sulfated polysaccharides present in the extracellular matrix or surface which constructs a steady chemokine gradient that ensures binding with its receptor [1].

## 2. Chemokine Receptor CCR6 and Its Ligand CCL20

CCR6 in *Homo sapiens* is identified by many synonymous codes: DCR2, DRY6, BN-1, CCR-6, CMKBR6, GPRCY4, STRL22, CD196, CKRL3, GPR29, CKR-L3, CC-CKR-6 or C-C CKR-6 [11]. CCL20 was identified as the sole known ligand to bind with CCR6 using CCL20 induced mobilization of calcium in K562 cells devoid of other receptors (CCR1-5) except CCR6 [1]. Five other chemokines (CCL2-5 and 17) did not bind to CCR6, although there is documented research stating CCL18 as a possible binding partner [12]. When CCL20 binds to CCR6, the receptor becomes internalized with its expression reduced on the cell surface [1]. Beta-defensins, a group of anti-bacterial peptides, have been shown to elicit chemotaxis through CCR6 although its activity on CCR6 still remains experimental [13].

CCL20, discovered using bioinformatics techniques is known by several names such as, macrophage inflammatory protein (MIP3α), Exodus-1 and liver and activation regulated chemokine (LARC). Th17 cells are known to express CCL20 but it is not expressed by the regulatory T cells or other T helper subsets [1]. CCL20 expression is markedly up regulated in the intestinal epithelial cells only as a response to intrusive or non-intrusive bacteria possessing flagella, and is labelled as an inflammation-inducing chemokine [14]. Cells linked to inflammation such as endothelial cells, neutrophils, Th17 cells, B cells, natural killer (NK) cells, dendritic cells, macrophages and Langerhans cells, reportedly express CCL20 [15].

CCR6 is naturally expressed in multiple tissues: maximally in the appendix, spleen, lymph nodes and pancreas and minimally in the thymus, colon, small intestine, fetal liver and testis [11]. CCR6 is upregulated by numerous leukocyte cohorts, such as B-cells, T-cells (specifically pro-inflammatory T_H_17 cells and immune regulatory T_reg_ cells), immature dendritic cells, NKT cells, innate lymphoid cell 3 and neutrophils [16]. 

The dominant role of CCR6 in inflammatory disease is underpinned by its influence on driving the T helper subset differentiation and maintaining leukocyte homeostasis. Naïve T helper cells resident in lymph nodes, upon antigen sampling will differentiate into its effector sub populations, T_H_17 and regulatory T_reg_ cells, T_H_1 and T_H_2, mediated by the prevailing cytokine environment and a host of other factors. However a critical factor which determines the development of T_H_17 and T_reg_ subsets evidently becomes the upregulation of CCR6 as both these cell sub types are known to be CCR6^+^ CD4^+^ T cells. Thus proliferation, migration and promoting pro-or anti -inflammatory effects of these helper sets might be primarily CCR6 dependent processes [17].

CD4^+^ T helper cells are the most important key players in organizing adaptive immunity. Naïve CD4^+^ CD45 RB ^high^ T cell populations, when activated by antigen presenting cells, differentiate into the mainstream T_H_1 and T_H_2 classical lineages. They regulate cellular immunity through their respective signature cytokine profiles, namely, IFN-γ, TNF-α and IL-1β in T_H_1 and IL-4, IL-5 and Il-13 in T_H_2. T_H_1 is pro-inflammatory by nature while T_H_2 mediates humoral and allergic responses [18]. T_H_1 and T_H_2 differ in immune function as well as migratory capability by expressing distinct chemokine e receptors which modulate selective recruitment into sites of inflammation. The other two T helper subsets, T_H_17 and regulatory T cells (T_regs_), specifically express CCR6 but not always by the T_H_1 or T_H_2 cells [19].

Research from different groups have conferred opposing roles to T_H_17 and regulatory T_reg_ cells in inflammatory disease [1]. IL-23 induced T_H_17 differentiation is known to promote inflammation and intriguingly T_H_17 also release CCL20, the chemokine ligand of CCR6. With either an autocrine or paracrine secretion of CCL20, T_H_ 17 cells also trigger a self-perpetuating cycle at inflammatory locations in a positive feedback loop. IL-17 production also induces TNF-α and IL-1β, pointing towards an inflammatory pathway involving the nuclear transcription factor NF-kB [20]. FoxP3^+^ bearing regulatory T_reg_ cells are primarily disease suppressive in function and promote disease resolution by downregulating inflammatory T cell proliferation and is inducible through TGF-β and IL-10 [21].

CCR6 expression on T_H_17 cells is induced through the cytokines, TGF-β, IL-6, IL-17, IL-21 and IL-23 as well as lineage- distinct, principal transcription factors RORγt and RORα [4]. CCL20 can be induced in a number of cell types by lipopolysaccharide (LPS) and also stimulated and up-regulated by the inflammatory cytokines IL-1α and TNFα during acute inflammation. CCL20 expression is constitutively up-regulated by other inflammation- inducing cytokines, namely, IFN-γ IL-1β, IL-17, IL-21 [22]. In contrast, IL-10, which is anti-inflammatory, dampens CCL20 expression indicating that this chemokine does not very well favor the accumulation of regulatory T cells particularly at inflammatory locations. IL-4, IL-22 and surprisingly, IL-23 have reported insignificant stimulatory effects on CCL20 expression [4].

CCR6 confers an antagonistic function in these T helper populations, the T_H_17 and Treg cells, although it is unknown what other factors are responsible for tipping the balance in favor of disease progression. CCR6-CCL20 axis remains the pivotal point which determines reciprocal generation of these two cell types but simultaneously highlights the importance of CCR6 in T cell migration.

A disrupted CCR6-CCL20 axis only leads to the development of inflammation because CCR6 is adequately expressed on regulatory T cell populations in the cure phenotypes. A study involving the transfer of “wild type” T_H_17 cells and CCR6 deficient T cells into a “Rag1^−/−^ severe combined immune deficient (SCID) mice” resulted in extreme intestinal inflammation and a subsequent reduction in T_H_17 and T_reg_ populations occurring [1]. CCR6 expression is deemed to be critical to T_reg_ cells than to T_H_17 cells, because this subset suppresses inflammatory T cell proliferation and promotes disease resolution. Research documented so far has assigned dual roles to CCR6 in inflammation and immune homeostasis [23].

## 3. CCR6 Signaling Pathway

CCR6 on leukocytes having bound to CCL20 initiates a signal transduction cascade via activation of the Gαi subfamily of heterotrimeric G proteins. Synonymous with most of the classical chemokine receptors, the downstream signaling of CCR6 initiates activation of calcium mobilization, phospholipase, and phosphatidylinositol 3-kinase, followed by ERK1/2 phosphorylation and actin polymerization [24].

**Figure 1:**
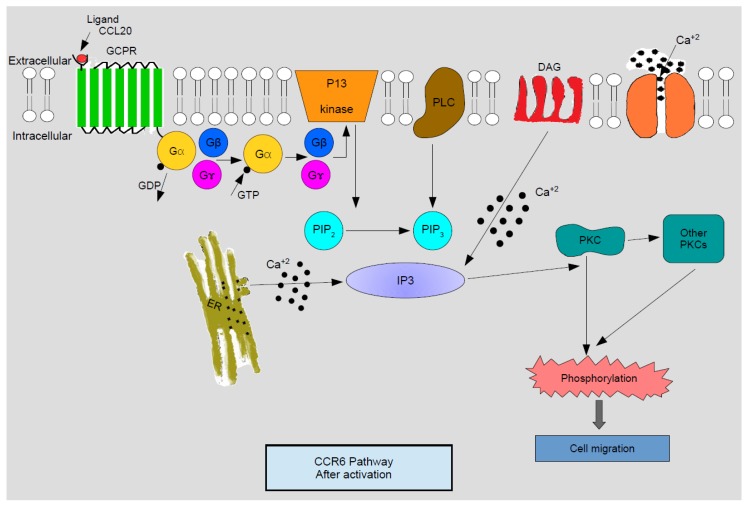
Schematic representation of C-C chemoattractant cytokine (chemokine) receptor 6 (CCR6) activation pathway. After binding with the ligand, G Protein Coupled Receptors (GPCR) are activated to release the Gβ and Gγ subunits which in turn activate PLC and PI3 kinase. PI3 kinase then converts PIP_2_ to PIP_3_ which activates calcium ion mobilization across IP_3_. IP_3_ with DAG stimulated by calcium ions activate PKC and other PKCs which trigger phosphorylation causing cell migration. Legend for abbreviations: Gα, Gβ, Gγ—Subunits of GPCR, PI3 kinase—Phosphoinositide 3 kinase, PLC—Phospholipase Cβ2, DAG—diacylglycerol, PIP_2_—Phosphatidylinositol 4,5-bisphosphates, PIP_3_—Phosphatidylinositol 4,5-bisphosphates 3, IP_3_—Inositol triphosphate, PKC—Protein kinase C, ER—endoplasmic reticulum, Ca^+2^—Calcium ions. Reproduced from [4]. Copyright 2003, CRC Press: Boca Raton, FL, USA.

Cell responses to chemokines include natural sensitivity to pertussis toxin, which is an indication that the G1 class proteins are annexed to the signaling pathway downstream of the receptor. As explained by Figure 1, activated G protein, subsequent to releasing guanosine di phosphate (GDP) and binding with guanosine tri phosphate (GTP), dissociates into Gβγ and Gα subunits. Gβγ then activates membrane-amalgamated phospholipase C-β2 (PLC) and phosphoinositide 3-kinase (PI_3_K). PI_3_K catalyzes phosphatidylinositol 4, 5 bi-phosphate (PIP_2_) into phosphatidylinositol 4, 5 bi-phosphate 3 (PIP_3_) which is next converted into inositol triphosphate (IP_3_). Concomitantly diacylglycerol (DAG) is activated by PLC. IP_3_ regulates the mobilization of calcium ions from intracellular depots and DAG acts in resonance with calcium to activate different isoforms of protein kinase C (PKC). Turned on PKC and various Ca—innervated protein kinases catalyze phosphorylation, thereby activating successive signaling episodes which end up in cell migration [4].

## 4. CCR6 and CCL20 in Health and Disease

**Figure 2:**
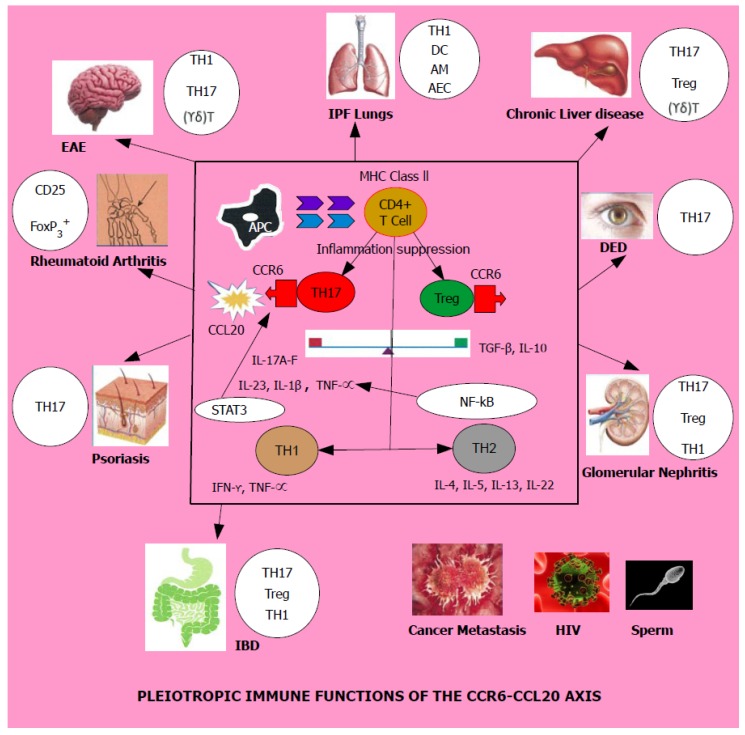
Schematic representation of the immunological impact of CCR6/CCL20 axis on multiple organs in the human body and the cells involved in promoting disease given next to each organ. EAE, IPF lungs, Chronic liver disease, DED, Rheumatoid arthritis, IBD, Psoriasis, Glomerular nephritis, HIV and Cancer metastasis are all diseases driven by the CCR6-CCL20 axis and disease progression leading to pathogenesis is promoted by T helper lymphocyte subsets, particularly T_H_17 and decrease in Treg cells. The factors which tilt the T_H_17-Treg balance influenced by transcription factors and cytokines in the tissue microenvironment is yet to be discovered. Legend for abbreviations: EAE—Experimental Autoimmune Encephalomyelitis, IPF lungs—Idiopathic Pulmonary Fibrosis lungs, DED—Dry Eye Disease, IBD—Inflammatory Bowel Disease, HIV—Human Immunodeficiency Virus, APC—Antigen Presenting Cell, MHC Class II—Major Histocompatibility Complex Class II, AM—Alveolar Macrophages, DC—Dendritic Cells, FB—Fibroblasts, AEC—Alveolar Epithelial Cells, CCR6—Chemokine receptor 6, CCL20—Chemokine ligand 20, IL—Interleukin, T_H_1, T_H_2, T_H_17, T_reg_—Regulatory T_reg_ cells—T helper lymphocyte subsets, (γδ) T cells—gamma delta T lymphocytes, NF-kB—nuclear factor kappa B, STAT3—signal transducer and activator of transcription 3. TNF-α—tumor necrosis factor-alpha.

CCR6 and CCL20 have been implicated as potential manipulators which create a significant impact on human health and disease. In normal health, this pair performs an immune tolerance role by up-regulating immune suppression. When confronted with an inflammatory stimulus FoxP3^+^ regulatory T_reg_ cells tend to proliferate aided by its cytokine milieu [25]. If this typical homeostatic function is disrupted, it is known to bring forth a marked increase of the T_H_1/T_H_17 axis thereby promoting adverse immunologic function of multiple systems culminating in a number of diseases, namely, sarcoidosis, idiopathic pulmonary fibrosis, chronic liver disease, experimental autoimmune encephalomyelitis, multiple sclerosis, rheumatoid arthritis, dry eye disease, psoriasis, glomerular nephritis, inflammatory bowel disease, HIV and an array of malignant cancers and their metastasis as shown by both Table 1 and Figure 2.

### 4.1. Lung

Adult human airways are not only a potential hub for harmful microbes, but are also exposed to allergens, pollutants, pulmonary emboli, complement deposition or autoantibodies, because the lungs are said to inhale around 10,000 L of air every 24 h. A typical response to these onslaughts by lung injury is the localized synthesis and release of distinctive chemokines, which amplify the inflammatory response. Inflammation is considered necessary in one way to defend, repair, protect against or subdue an infection. Thus, activating leukocyte chemotaxis is vital to the development of antimicrobial host defense [4].

Research from Facco et al. on pulmonary sarcoidosis had revealed CCR6 expression on T_H_1 cells. CCR6 was co-expressed on alveolar macrophages in patients of sarcoidosis and alveolitis along with CXCR3 and CXCR6. CCR6^+^ T cells infiltrated into the lung interstitial tissue and were responsive to CCL20, CXCL10 and CXCL16. This observation amply demonstrates that T cells bearing CCR6 act in a coordinated manner with ligand and inflammatory cytokines produced by T_H_1 during alveolitic disease [25]. Further, CCR6 possesses the capability to recruit antigen-presenting immature and mature dendritic cells (DC) and macrophages to sites of inflammation on the alveolar epithelium [26].

CCL18, released by alternatively activated macrophages, induced collagen synthesis by human lung fibroblasts, indicating CCR6 as the receptor for CCL18, reported from a screened phage-display library. Staining lung tissue indicated CCR6 presence on alveolar epithelial cells II (AEC-II) and fibroblasts in idiopathic pulmonary fibrotic (IPF) lungs that blocks airways, but not in tumor-free areas of squamous cell carcinoma patients. It inferred a role for CCR6-CCL18 involving fibroblasts in human lung disease [12].

In contrast, gene delivery of human CCL18 to the lungs of wild type (WT) mice induced infiltration of T lymphocytes to the lungs, but the expression of CCR6 was found to be below 5% in the lymphocyte population. In the lungs of CCR6 knockout mice, CCL18-driven T lymphocyte trafficking was reduced but not completely halted. These observations, made in vivo in mice, concluded that CCR6 was not required for CCL18-induced changes, and that CCR6 is not the principal receptor for CCL18 in this animal model [27].

### 4.2. Kidney

Glomerulonephritis is characterized by tissue damage caused due to T cell trafficking into the kidney. Chemokines modulate the migration of T lymphocytes to sites of inflammation. Renal FoxP3^+^ regulatory T cells (T_reg_) and IL-17 releasing T_H_17 cells were shown to upregulate CCR6 while IFN-γ releasing T_H_1 cells were CCR6 negative. T_regs_ and T_H_17 subsets displayed migratory capability towards CCL20 which was markedly high in renal biopsies of experimental murine nephritis. T cell recruitment was followed by pathogenesis in the kidney with albuminuria, leading to loss of renal function [28]. Nephritic mice deficient in CCR6 demonstrated extreme renal damage and high mortality in comparison to the wild type, due to less accumulation of T_reg_ cells and T_H_17 cells and not that of the T_H_1 type. This suggests an imbalance of T_reg_/T_H_17 paradigm associated with CCR6 mediation exists in the kidneys too, similar to the gut. Reintroduction of WT T_reg_ had offered protection to CCR6 knockout mice against severe renal injury, confirming that CCR6 promotes the recruitment of both T_H_17 and regulatory T reg cells to the kidney whereas a decrease in T_regs_ in the presence of T_H_1 response produced aggravated disease [29]. T_regs_ have been implicated in maintaining tolerance to autoimmune renal disease, thereby lowering renal inflammation, and in preventing allogenic responses in renal transplantation [30].

CCR6 and CCL20 are reportedly involved in recruiting T and B cells to kidney nodules during chronic inflammation in individuals. The CCR6/CCL20 mRNA expression quantified in renal biopsies of various nephropathies had revealed this cognate chemokine receptor was expressed in a number of kidney-associated cells: CD20^+^ B cells, CD3^+^ T cells, tubular epithelial cells and the peritubular and glomerular capillary endothelial cells although the functional role of CCR6 in the renal endothelium had not been assessed. Similarly to CCR6-CCL20 acting as a mediator in the modelling of gut-associated lymphatic tissue, it is postulated that the nodular infiltrates in the kidney are also formed in a CCR6-dependent manner [31].

### 4.3. Liver

Chronic liver injury results from hepatic inflammation, leading to organ fibrosis. Intrahepatic increase in CCR6 and CCL20 expression was observed in patients with chronic liver disease compared to healthy controls. It demonstrated that this chemokine receptor ligand pair may contribute to the migration of gamma-delta (γδ) T cells, T_H_17 and regulatory (T_reg_) cells to sites of inflammation. CCR6 was explicitly required by IL-17 expressing γδ T cells to gather in the injured liver and promote disease resolution. Immunohistochemistry revealed accumulation of mononuclear cells bearing CCR6 induced by CCL20 secretion of hepatic parenchymal tissue in clinical liver disease. Compared to the WT, CCR6 knockout mice developed more acute fibrosis with enhanced immune cell infiltration to the liver [32,33].

### 4.4. Brain

T_H_17 is strongly associated with autoimmune diseases, as demonstrated by pre-clinical studies in rheumatoid arthritis and multiple sclerosis. Neutralizing IL-17 as well as transfer of T_H_17 lacking CCR6 receptors had markedly inhibited experimental autoimmune encephalomyelitis (EAE). Apart from autoimmune promoting, pro-inflammatory function of T_H_17 it is also known to bring about disease resolution. Chemokines and adhesion molecules activate T cells, propelling them to migrate towards the central nervous system (CNS). The choroid plexus constitutively express CCL20 and is thought to act as an entry point for CCR6 expressing CD4^+^ T cells. EAE in animal models is used to study multiple sclerosis, which is a demyelinating inflammatory disorder of the CNS and infiltrating T cells contribute to its pathogenesis. Effector T_H_17 and T_H_1 subsets are found in multiple sclerosis lesions along with the expression of cytokines IL-17 and IFN-γ. CCR6 demonstrates a critical aspect in the entry of T_H_17 which is said to induce EAE in the CNS. CNS-infiltrating cells, when analyzed directly for CCR6 expression, have revealed that in EAE, T_H_1 cells are in excess of T_H_17 CD4^+^ and both subtypes however, expressed CCR6 [34,35].

Cerebral ischemia or stroke is ranked the second globally most common cause of death and is a much-debilitating neurological disease condition. Immune-mediated tissue damage occurs in the first few days of suffering a stroke and is mainly attributed to brain-infiltrating, IL-17 releasing, γδ T cells which are largely positive for the chemokine receptor CCR6 as they trigger a highly conserved immune reaction. In a model of experimental stroke, genetic deficiency in *Ccr6* was associated with diminished infiltration of natural IL-17 releasing γδ T cells and a significantly improved neurological outcome, outlining the role CCR6 plays in pro-inflammatory immune cell chemotaxis to inflamed sites in the brain [36].

### 4.5. Eye

T_H_17 cells are the principal effector cells causing inflammation in dry eye disease (DED), an immune inflammatory condition affecting the ocular surface that can even lead to corneal perforation. Local neutralization of CCL20 with antibodies administered sub-conjunctively to DED mice had decreased T_H_17 cell permeation into the ocular surface producing improvement in clinical signs, suggesting that CCR6 interaction with CCL20 could direct the passage of T_H_17 cells in the eye. Inhibition of the CCR6/CCL20 axis is therefore projected as a novel therapeutic mechanism to treat this condition [37].

### 4.6. Skin

The skin disorder atopic dermatitis (AD) is identified by a deficiency of keratinocytes in the skin, which produces less CCL20, and similarly such patients also display a reduction in the expression of CCR6, which leaves them exposed to viral infections leading to eczema herpeticum (ADEH) or eczema vaccinatum (EV). A population-based study of European and African descent had recorded single nucleotide polymorphism (SNP) in CCL20 in native Europeans significantly associated with AD, suggesting that variants in CCL20 and CCR6 are highly relevant to AD and increase the risk of severe viral complications in this skin disease [38].

Psoriasis is a commonly occurring autoimmune skin disease that involves T_H_17 associated signaling pathways. CCR6 deficient mice had failed to develop psoriasiform dermatitis in skin following IL-23 injections, because IL-23 is a growth and differentiation factor of T_H_17 cells and hence a typical driver of T_H_17 mediated inflammation, validated by previous research showing that recombinant IL-23 injections given to mice skin results in psoriasiform dermatitis that mimics human psoriasis in as short a period as 5 days. A more recent experimental model has documented that dermal CCR6^+^ T_H_17 cells are sustained by IL-23 released from dendritic cells and these T_H_17 populations release IL-22 to stimulate epidermal hyperplasia through signal transducer and activator of transcription 3 (STAT3) mediated mechanisms in the human skin. Additionally, positive feedback was provided by epidermal and dermal production of CCL20, potentially recruiting more CCR6 expressing T cells or antigen presenting cells into inflamed psoriatic skin. Inhibition of CCR6 has been suggested to provide a possible therapeutic pathway to cure this disease [39,40].

### 4.7. Joints

Rheumatoid arthritis causes chronic inflammation of the joints where chemokines regulate infiltration of synovial fluid by inflammatory cells. This autoimmune disease is characterized by the increased release of CCL20 and the buildup of CCR6 bearing mononuclear T cells in the joints. An arthritis-induced study model of CCR6^−/−^ mice had not exhibited any clinical signs consistent with disease compared to WT controls, but revealed that CD4^+^ T cells, T_H_17 cells and CD25 FoxP3^+^ regulatory T cells showed up-regulation of CCR6 with RANKL, which contributed towards disease, particularly osteoclastogenesis. A possible role in pathogenesis is thus highlighted in CCR6 in promoting inflammation at the joints [41,42,43]. *Ccr6* single nucleotide polymorphisms (SNPs) have demonstrated diminished basal and ligand induced Gαi protein signaling which predisposes individuals to diseases such as rheumatoid arthritis [44].

### 4.8. Gonad

Capacitated human sperm is said to exhibit a directional movement towards CCL20 having the CCR6 receptor localized in the tail and a recent study revealed modifications in motility parameters of spermatozoa in the presence of chemokines. In non-inflammatory conditions, chemokine receptor/ligand interactions within the reproductive tracts of the both sexes are known to promote sperm motility and chemotaxis. Physiological reactions are thus mediated by CCR6 ligands in the male genitourinary system which extends beyond an inflammatory response, and this observation underpins its significance in clinical reproduction and also possibly in contraception [45].

### 4.9. Gut

Animal models of the past and present have identified: (i) genetic predisposition; (ii) the composition of associated microbiome; (iii) breakdown of innate immune barriers—disruption of the mucosal barrier due to decreased mucin synthesis, dysfunctional Toll-like and Nod-like receptor mediated pathways leading to increased pathogenicity, endoplasmic reticulum (ER) stress mediated apoptosis; (iv) deregulated adaptive immunity; and (v) a plethora of environmental factors, as multiple causes responsible for inflammatory disorders in the gastrointestinal tract (GI) and disruption of the CCR6/CCL20 axis, also as a significant contributing factor [46,47,48,49].

GWAS have confirmed *Ccr6* as a risk allele of GI tract infections, among the 200 or so susceptibility loci already identified. It confirms that infectious diseases of the gut exhibit gene dependency and also gives prominence to the CCR6/CCL20 axis as a potential risk factor which determines disease outcome. T_H_17 cells are directed to the small intestine by CCR6 upon immune induction and not only T_H_17, but also FoxP3^+^ regulatory Tregs are upregulated, given the fact that CCR6 performs dual functions with regards to these two helper T subsets in gut associated lymphoid tissue (GALT). Accumulation of T_H_17 cells in the spleen and bone marrow in CCR6 deficient mice showed they were unable to migrate due to the absence of this receptor, and hence produced less intestinal inflammation. This fact further supports its role in directing immune cell movement in the gut and confirms that T_H_17 plays a pro-inflammatory role in intestinal disorders [50,51,52].

Intestinal microbiome which comprises of around 100 trillion cells is important for: (i) colonization and maintenance of immune cells; (ii) T_H_17—Treg balance in the gut; and (iii) protection against intestinal pathogens, evidenced by a reduction in T_H_17 and elevated Treg populations in mice given: (i) antibiotics; and (ii) bred in germ-free conditions. The disease outcome therefore primarily depends upon the CCR6-CCL20 axis, with microbiota featuring as another additional contributor [53,54].

Inflammatory bowel disease (IBD), which is an autoimmune GI tract disorder, consists of two clinical variants, Crohn’s disease and Ulcerative colitis. A Ccr6 knockout murine models had displayed: (i) smaller Peyer’s patches; (ii) reduced sub epithelial domes; (iii) absence of isolated lymphoid follicles; (iv) reduced intestinal M cell numbers; (v) increased resistance to bacteria which enters through M cell conduits; (vi) marked elevation in the number of T_H_17 cells in the spleen and lymph nodes; (vii) Reduced migration to inflamed sites and less suppressive capabilities of Treg cells; (viii) moderate and severe disease in DSS and TNBS induced colitis respectively; and (ix) transfer of naïve T cells to Rag2^−/−^ mice resulting in aggravated disease [55,56,57]. SNPs in *Ccr6* have been reported to predispose individuals to Crohn’s disease [44].

### 4.10. Cancer

Chemokines are utilized by cancer cells to directly invade the lymphatic system and spread via blood, as well as determine the location of metastatic growth of various tumors. CCL20 has been reportedly expressed in varied human cancer types, such as melanoma, adenocarcinoma, hepatocellular carcinoma leukemia, lymphoma, prostate cancer, colorectal, oral and lung squamous cell carcinoma and pancreatic carcinoma (PCA) [58,59]. The CCL20/CCR6 system has been demonstrated within pancreatic cancer cell lines and PCA-associated tissues. The stimulation of PCA cells expressing CCR6 with CCL20 had constitutively triggered cell proliferation, tendency to migrate and invasion of tissues indicating that CCL20 could act using mechanisms of autocrine and paracrine secretion. Recent studies have identified matrix metalloproteinase production to up-regulate CCL20, which promotes pancreatic tumor cell movement and their metastatic invasion [58].

A new interesting theme that involves chemokine receptor function is that chemokine receptors are equipped to drive cancer metastasis. Similar to CCR7 being useful for the movement of T cells within lymph nodes, when chemokine receptors are expressed in tumor cells, they may direct organ-targeted tumor metastasis. Precise signals which exist on the tumor cell surface, along with the establishment of chemotactic gradients, will allow them to migrate to metastatic destinations of numerous cancers. Various chemokine ligands have been strongly identified in directing metastasis into lymph nodes [59,60,61].

The chemokine superfamily is said to provide a useful target to find new important drugs because G protein coupled receptors (GPCR) are ideal for testing small molecular inhibitors, and therefore many best-selling anti-cancer therapeutics in the market today have targeted GPCR [62,63]. CCR6 inhibition in patients undergoing surgical treatment or clinical therapy has been proposed to be important to prevent liver metastasis of cancer, by a study of BALB/c mice. They showed overexpression of functional CCR6 and CCR7 on metastatic tumor cell lines obtained from the liver. Cell migration was studied employing green fluorescent protein-labelled metastatic cells and CCR6 mRNA was measured by RT-PCR. Results displayed preferential colonization of liver-sorted tumor cells when pre-incubated with the CCR6 ligand, which is constitutively expressed by hepatocytes. This demonstrates that CCR6 directs and drives the mechanisms of chemotaxis, commonly adopted by malignant cancers when metastasizing to the liver [33]. Mutations in *Ccr6* also have been associated with a case of mucosa-associated lymphoid tissue (MALT) lymphoma [64].

### 4.11. HIV

Preferential infection by HIV of CCR6^+^ T_H_17 cells in vitro has been described in a study which had cultured both T_H_1 and T_H_17 cells obtained from peripheral blood of healthy individuals in the presence of activated IL-1β and IL-23. IL-23 was added to promote the expansion of T_H_17 cells. Infection by HIV had produced negligible effects on T_H_1 whilst causing a significant reduction in T_H_17 cells, increased infection of T_H_ 17 cells and cell death. It reportedly demonstrated a possible role for CCR6 in the internalizing of the virus within T helper populations [65]. In a more recent observation in relation to HIV infection of T_H_17 cells, CCR6 was presented as a weak alternative sub-receptor alongside the receptors CXCR4 and CCR5, which may provide an entry route to HIV invading the T helper cell subsets. This fact suggests that preferential infection of T cells takes place without CCR6 mediation. Yet, the importance of the CCR6/CCL20 axis is by no way diminished in HIV pathogenesis because this receptor- ligand pair is deemed to be actively recruiting T_H_17 cells and DCs to infection sites, thus helping the virus to propagate to other locations of the body [66]. Envelope surface glycoprotein gp120 is known to significantly promote the CCR6 expression on human B cells [11].

### 4.12. Obesity and Diabetes

The CCR6-CCL20 alliance is said to promote inflammation, leading to pathophysiological malignancies such as obesity and diabetes. Minor inflammation of the adipose tissue has been linked with obesity and is tactically driven via the CCR6-CCL20 axis. Adipose tissue lymphocytes expressing CCR6 have demonstrated chemotactic migration towards mature adipocytes which upregulate the chemokine ligand CCL20. Strongly enhanced CCL20 expression by adipocytes displayed a positive correlation with the body mass index (BMI), in visceral adipose tissue compared to the subcutaneous fat layers [67].

Increased leukocyte streaming into pancreatic islets causes inflammation in the beta cell mass influencing apoptosis and dysfunction. A study by Burke et al. had identified elevation of CCL20 levels in pancreatic beta cells induced by the transcriptional factor nuclear factor kappa B (NF-kB). They suggested intervening at the NF-kB –mediated chemokine upregulation, to disrupt the CCR6-CCL20 axis, which could become a therapeutic mechanism to negate inflammation-triggered dysfunction seen in both obese and diabetic individuals. T cell immunity is suppressed by the activation of type III histone deacetylase Sirtuin 1, which is known to regulate cellular processes via the SIRT1 gene. Resveratrol is a Sirtuin-1 activator which demonstrated therapeutic efficacy in a Nucleotide-binding and oligomerization domain (NOD) mouse model of type 1 diabetes. Resveratrol-treated mice exhibited a significant decrease in *Ccr6* in a gene array analysis, correlating with decreased migration in CCR6^+^ macrophages and IL-17 producing cells into the pancreas from pancreatic lymph nodes [68,69].

## 5. Conclusions

The CCR6/CCL20 axis thus demonstrates a vital role in determining the disease outcome of inflammatory disorders and cancer metastasis in multiple organ systems in the human body. Inhibition of the CCR6/CCL20 axis is expected to open up new vistas for discovering novel therapeutics in the treatment of human disorders, and a variety of therapeutic antibodies have already been made against the chemokine receptor 6. Effective blocking of the CCR6 signal transduction pathway using novel biochemical interventions might prove to be a useful therapeutic strategy in regulating pro-disease inducing cell chemotaxis within inflammatory microenvironments. Most of the research has been centered on animal models which do not depict the true clinical picture, therefore there remains an urgent need for the evaluation of this chemokine receptor 6 and its ligand pair in expanded in vivo and ex vivo clinical studies.

## Figures and Tables

**Table 1:** Organ systems and the diseases in which CCR6/CCL20 axis is operative along with the cell types involved in the disease progression which express CCR6.

Organ	CCR6/CCL20 Axis	Cell Types Involved	Disease Produced	References
Lung	Operative	Fibroblasts, AEC, DC, T_H_1, AM	IPFL, Sarcoidosis	[12,25,26]
Liver	Operative	T_H_17, T_reg_, (γδ) T cells	Chronic liver disease	[32,33]
Kidney	Operative	T_H_1, T_H_17, T_reg_	Glomerular nephritis	[28,29,30,31]
Brain	Operative	T_H_1, T_H_17, (γδ) T cells	EAE, Stroke	[34,35,36]
Eye	Operative	T_H_17	Dry eye disease	[37]
Skin	Operative	T_H_17	Psoriasis	[39,40]
Joints	Operative	CD25^-^FoxP3^+^	Rheumatoid arthritis	[41,42,43]
Gut	Operative	T_H_17, T_reg_, T_H_1	IBD	[1,50,51,52]
